# Ketamine for acute pain after trauma: the KAPT randomized controlled trial

**DOI:** 10.1186/s13063-022-06511-6

**Published:** 2022-07-27

**Authors:** Thaddeus J. Puzio, James Klugh, Michael W. Wandling, Charles Green, Julius Balogh, Samuel J. Prater, Christopher T. Stephens, Paulina B. Sergot, Charles E. Wade, Lillian S. Kao, John A. Harvin

**Affiliations:** 1 Department of Surgery, McGovern Medical School at UTHealth, The University of Texas, Houston, TX USA; 2 The University of Texas Medical Center at Houston, Houston, TX USA; 3 Center for Clinical Research and Evidence Based Medicine, McGovern Medical School at UTHealth, Houston, TX USA; 4 Department of Anesthesia, University of Arkansas for Medical Sciences, Little Rock, USA; 5 Department of Emergency Medicine, The University of Texas Medical Center at Houston, Houston, USA; 6 Department of Anesthesiology, McGovern Medical School at UTHealth, Houston, TX USA; 7 Center for Translational Injury Research, Department of Surgery, McGovern Medical School at UTHealth, Houston, TX USA

**Keywords:** Ketamine, Multimodal pain control, Analgesia

## Abstract

**Background:**

Evidence for effective pain management and opioid minimization of intravenous ketamine in elective surgery has been extrapolated to acutely injured patients, despite limited supporting evidence in this population. This trial seeks to determine the effectiveness of the addition of sub-dissociative ketamine to a pill-based, opioid-minimizing multi-modal pain regimen (MMPR) for post traumatic pain.

**Methods:**

This is a single-center, parallel-group, randomized, controlled comparative effectiveness trial comparing a MMPR to a MMPR plus a sub-dissociative ketamine infusion. All trauma patients 16 years and older admitted following a trauma which require intermediate (IMU) or intensive care unit (ICU) level of care are eligible. Prisoners, patients who are pregnant, patients not expected to survive, and those with contraindications to ketamine are excluded from this study. The primary outcome is opioid use, measured by morphine milligram equivalents (MME) per patient per day (MME/patient/day). The secondary outcomes include total MME, pain scores, morbidity, lengths of stay, opioid prescriptions at discharge, and patient centered outcomes at discharge and 6 months.

**Discussion:**

This trial will determine the effectiveness of sub-dissociative ketamine infusion as part of a MMPR in reducing in-hospital opioid exposure in adult trauma patients. Furthermore, it will inform decisions regarding acute pain strategies on patient centered outcomes.

**Trial registration:**

The Ketamine for Acute Pain Management After Trauma (KAPT) with registration # NCT04129086 was registered on October 16, 2019.

## Background and rationale

In a learning trauma care system, optimal patient outcomes are achieved through continuous learning and improvement facilitated by rapid evidence generation and innovation, implementation of evidence into care delivery, and refinement [1]. As an example of a learning trauma care system, the Red Duke Trauma Institute (RDTI) has made iterative changes to minimize opioids through implementation of a multi-modal pain regimen (MMPR). In 2013, a MMPR was implemented consisting of five different classes of pain medication given in a scheduled fashion with oral opioids available as needed. This original MMPR resulted in an approximately 31% reduction in average morphine milligram equivalents (MME) per patient day and a reduction in patient-reported pain scores [2]. In order to assess the effectiveness of less expensive alternatives, the Multimodal Analgesic Strategies in Trauma (MAST) trial was conducted, randomizing patients to the original MMPR versus a generic, widely available MMPR. The generic MMPR resulted in lower in-hospital opioid exposure and opioid prescribing without a clinically significant increase in pain scores [3]. The MAST MMPR replaced the original MMPR as usual care locally.

Despite these improvements, short-term opioid administration for acute pain continues to be associated with a small but substantial risk of persistent opioid use [4]. In the MAST trial, despite the reduction in opioid exposure, 62% of patients were discharged with an opioid prescription. In both the MAST trial and a multicenter observational study, in-hospital opioid exposure was highest within the first 72 h after admission [3, 5]. While new medications and non-medicinal interventions to minimize opioid exposure are needed, patients are still arriving to trauma centers with acute pain that must be treated. Thus, continued refinement and improvement of our MMPR is needed.

Ketamine is a Federal Drug Administration approved drug that is used for general anesthesia and for procedural sedation. Ketamine can also be used for treatment of chronic pain and post-traumatic stress disorder (PTSD) [6, 7]. More recently, there has been increased interest in sub-dissociative doses of ketamine for the treatment of acute pain. There is evidence that ketamine is effective in in bolus form for treating acute pain in the pre-hospital and emergency room setting but data for use in acute traumatic pain is limited [8–10]. The aim of this study is to determine the effectiveness of continuous infusions of ketamine in the acutely injured trauma patient.

## Objectives

The Ketamine for Acute Pain after Trauma (KAPT) trial is a single-center, parallel-group, randomized, controlled comparative effectiveness trial of our MMPR to our MMPR plus a sub-dissociative ketamine infusion for the first 72 h after admission. We hypothesize that the addition of ketamine will result in lower opioid exposure in injured patients compared to our generic MMPR alone as evidenced by lower average oral MME per day. The objective of this study are:

1. To ascertain the effectiveness of ketamine for treatment of acute pain by evidence of reduced opioid consumption
2. To ascertain the effectiveness of ketamine for treatment of acute pain by evidence of self-reported patient pain scores

## Trial design

**Fig. 1:**
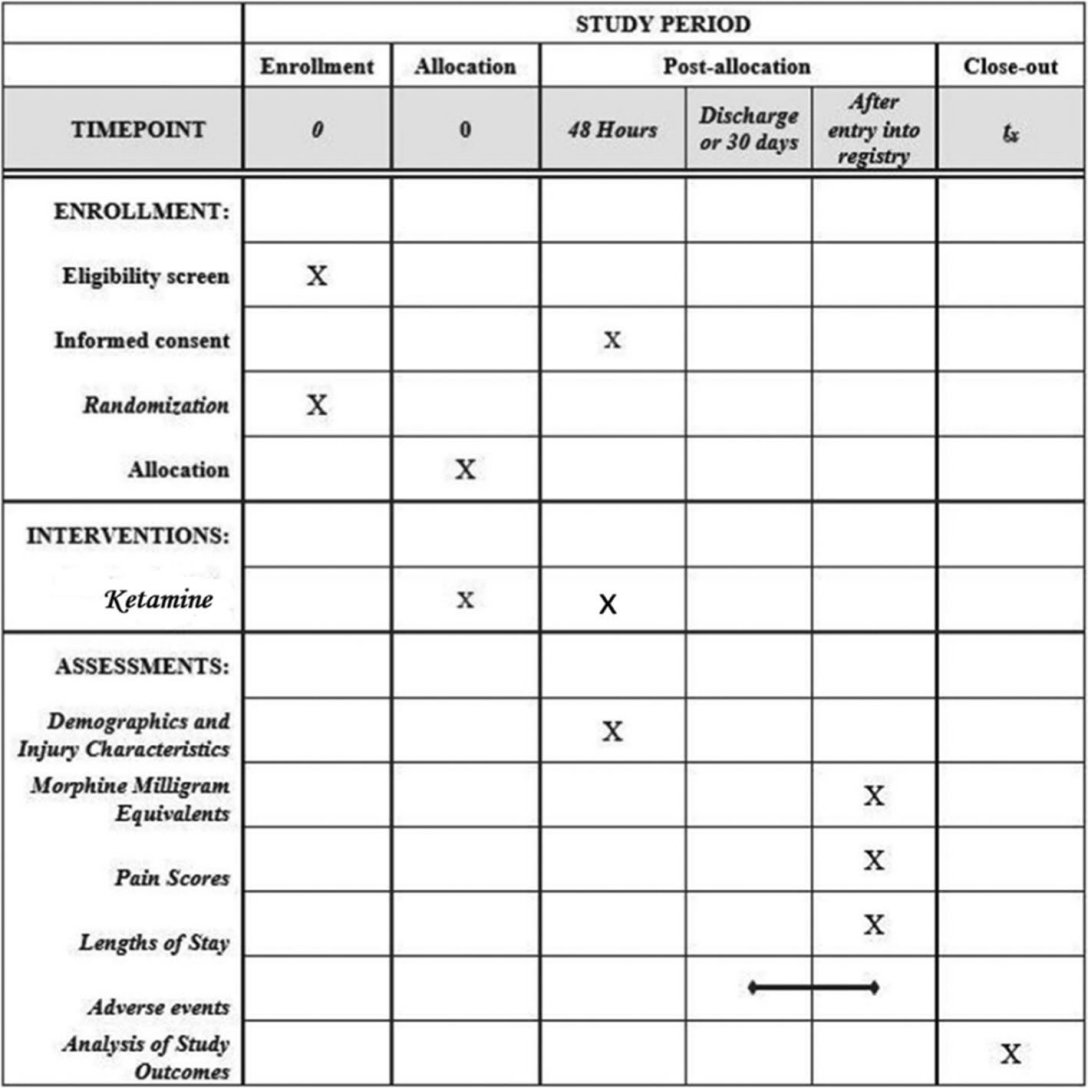
Standard Protocol Items: Recommendations for Interventional Trials Diagram. The figure details timing of enrollment activities, intervention allocation, and assessments of outcomes during the course of the clinical trial

The trial is registered at ClinicalTrials.gov (Identifier: NCT04129086) and was designed in accordance with the Standard Protocol Items: Recommendations for Interventional Trials 2013 guidelines (Fig. 1). This manuscript with adherence to SPIRIT reporting guidelines [11]. Ketamine is a commonly used medication at our institution, but to facilitate implementation, an electronic order set was created that standardized the dosing regimen and the drug was stocked in the emergency department pharmacy. Additionally, nursing education was performed for nurses unfamiliar with ketamine.

## Protocol amendments

There have been no protocol amendments.

## Study setting

This study is being conducted at the RDTI at the Memorial Hermann -Texas Medical Center, which is one of two level 1 trauma centers in Houston, Texas. It is a high-volume, teaching hospital for the McGovern Medical School at the University of Texas Health Science Center at Houston.

## Eligibility criteria

Inclusion criteria include the following: all adult patients (≥ 16 years of age) admitted to the adult trauma service at the intermediate or intensive level of care. Exclusion criteria include the following: pregnant women, prisoners, and those not expected to survive. Additionally, patients with the following contraindications to ketamine are excluded: allergy to ketamine, poorly controlled hypertension, cardiac arrhythmia (including atrial fibrillation), congestive heart failure, unstable coronary artery disease or recent myocardial infarction, cirrhosis, dementia, movement disorder (e.g., Parkinson’s), or seizure disorder. Additionally, for those unable to provide past medical history, patients are excluded if they have arrhythmia on EKG, are older than 65 years, present after fall from standing, or have median sternotomy scar. Patients in the ketamine arm may drop out of the treatment group if ketamine is stopped prematurely due to medication side effects (otherwise unexplained tachycardia, arrhythmia).

## Informed consent

We obtained a waiver of consent to randomize patients due to the following:

1. The intervention is minimal risk; the ketamine dosing proposed is already being used by prehospital personnel, emergency room physicians, and trauma surgeons at similar or higher doses.
2. The research could not be practicably carried out without the waiver; due to the acute clinical status of the trauma patient population (intubation, intoxication, severe pain, emotional stress), *truly informed* consent cannot be obtained in the vast majority of patients (or their legally authorized representative) before the ketamine would be given.
3. The waiver would not adversely affect the rights and welfare of the subjects; patients often receive opioids and/or ketamine in the Emergency Department acutely without their knowledge or written consent, they simply want pain control. We will be providing pain control and rescue opioids would not be withheld from the intervention group.

Once the patient is randomized, a member of the trauma research team attempts to contact either the patient or legally authorized representative to obtain consent for using Protected Health Information, to return to perform discharge surveys, and to contact after discharge for post-discharge surveys. Consents are obtained by trained research personnel and patients are given written study information. If, after 5 days, the patient remains unable to self-consent and no LAR is available to consent, the consent is waived and data included. Additionally, if the subject does not survive following the traumatic injury or is discharged from the hospital before the study team is able to obtain consent, their information is included in data analysis.

## Interventions

### Usual care group

Trauma patients at the RDTI are initially prescribed with an opioid-minimizing MMPR informed by the MAST trial [3]. This regimen consists of four different classes of medications given in a scheduled fashion: acetaminophen, naproxen, gabapentin, and lidocaine patch(s). Tramadol or oxycodone are administered as needed for additional pain control. The bedside provider is allowed to adjust the pain regimen as needed.

### Ketamine plus usual care group

Patients in the ketamine group receive sub-dissociative ketamine for a maximum of 72 h in addition to MMPR (Table 1). Ketamine is administered as a bolus dose of 0.35 mg/kg and then given as a continuous infusion of 0.1–0.25 mg/kg/h. This dosing was determined based on the American Society of Regional Anesthesia and Pain Medicine, the American Academy of Pain Medicine, and the American Society of Anesthesiologists consensus guidelines [12]. Ideally, the infusion is continued for a minimum of 24 h and a maximum of 72 h; however, the bedside clinician is free to alter the pain regimen as seen fit.

**Table 1:** Treatment groups

Group	Ketamine Infusion Group	Usual Care
Drug dose	*Bolus:* 0.35 mg/kg *Infusion:* start 0.15 mg/kg/hr; titration range is 0.1–0.25 mg/kg/hr	None
Duration	*24 (minimum) to 72 (maximum) hours after admission and each subsequent major surgery*	n/a
Usual Care	• Acetaminophen 1,000 mg PO q6 hours • Naproxen 500 mg PO q12 hours • Gabapentin 300 mg PO q8 hours • Lidocaine patches Opioids as needed for additional pain control	• Acetaminophen 1,000 mg PO q6 hours • Naproxen 500 mg PO q12 hours • Gabapentin 300 mg PO q8 hours • Lidocaine patches Opioids as needed for additional pain control
Timing	Ketamine drip to begin as early as possible: • Hemodynamically stable patients – start immediately (ideally in ED prior to CT scan) • Hemodynamically unstable patients – start once stabilized	

*Mg* milligram, *kg* kilogram, *hr* hour

The treatment is initiated in the emergency department. In both treatment groups of this pragmatic trial, adjustments may include withholding or adjusting the dosage of MMPR medications due to comorbidities such as kidney or liver disease, withholding a medication due to interactions with other medications, and de-escalation of medications once adequate pain control is achieved. In both regimens, oral medications are also available as needed for breakthrough (moderate and severe) pain—oxycodone and/or tramadol. Although their use is discouraged in general clinical practice at our institution, intravenous opioids can be administered as needed.

## Outcomes

The primary outcome is opioid exposure defined as average MME per patient per day. MME/day was calculated by converting all sources of opioids during the hospital stay, including emergency department and operating room, to a single MME value using a standardized conversion chart and dividing by length of hospital stay (Table 2). MME/day was chosen as the value can be easily calculated by other centers with which to compare their patients’ opioid exposure and the value accounts for length of stay.

**Table 2:** MME conversion

	Conversion factor
**Oral opioids**	
Codeine (mg)	0.15
Tramadol (mg)	0.1
Hydrocodone (mg)	1
Oxycodone (mg)	1.5
Methadone (mg/day)	
1–20	4
21–40	8
41–60	10
> 61–80	12
Morphine (mg)	1
Hydromorphone (mg)	4
**Transdermal opioids:**	
Fentanyl (μg)	2.4
**Intravenous opioids:**	
Morphine (mg)	3
Hydromorphone (mg)	15
Fentanyl (μg)	0.2

Morphine milligram equivalents (MME) will be calculated by converting consumed opioids to MME using above conversion factors

Secondary opioid exposure outcomes include total MME and opioid prescribing at discharge. Other secondary outcomes include pain scores, (Behavioral Pain Score (BPS), Numeric Rating Score (NRS), and/or Defense and Veterans Pain Rating Scale (DVPRS), potential opioid-related complications (delirium, unplanned intubation, and unplanned admission to an intensive care unit), and ketamine-related information (time to infusion initiation, duration of infusion, etiology for infusion cessation). Additional secondary outcomes include use of regional anesthesia, lengths of stay, and hospital costs. Patient requests to discontinue ketamine infusions and incidence of delirium are also being recorded.

At 6-month follow-up, patients are contacted to assess for the presence of persistent post-traumatic pain and persistent opioid use. Additionally, at that time, patient-centered outcomes including quality of life will be assessed using Euro-QOL EQ-5D-3L [13].

## Sample size

Due to the uncertainty of an exact treatment effect of ketamine in our patient population and the potential for contamination of the control group if the trial is continued for a prolonged length of time, we plan to perform the largest feasible study over 18 months. Over a sample period of 6 months (November 2018–April 2019), manual review of admission to the trauma service revealed 302 potentially eligible patients. Extrapolating this data, we expect 906 potentially eligible patients over the 18-month trial period. Given the emergent nature of the intervention, general difficulties enrolling severely injured patients, and several concomitant randomized clinical trials, we conservatively estimate enrolling 40% of potentially eligible patients.

We anticipate recruiting approximately 362 patients into the current trial. In an attempt to enhance participant enrolment and reach target sample size, all admitted patients are screened daily by research assistants for possible enrollment. Based on its skewed distribution, MMEs per day are postulated to be gamma distributed (MMEs need not be integer values). Using the 50th and 75th percentiles, MME = 45 and 67 respectively, from our previous data, we estimate the control condition as following the distribution ~Gamma (shape = 2.7784923, scale = 18.343307). Further, assuming that the ketamine condition results in a 20% decrease in MMEs (50th and 75th percentiles MME = 36 and 53.6 respectively), we assume the observed data for this condition is distributed ~Gamma (shape = 2.7784923, scale = 14.674646). We assume that clustering within unit induces a substantial intra-cluster correlation (ICC) = 0.2. Finally, we stipulated that a 75% chance that the ketamine treatment reduces MMEs by at least 15% relative to treatment as usual would constitute sufficient evidence to warrant a larger clinical trial. Assuming the previously stated sample size, effect, ICC and decision rule, *K* = 1000 Monte Carlo simulations suggest there is a > 99% chance of observing this effect.

## Sequence generation

Randomization is performed at admission while in the emergency department in a 1:1 allocation and stratified by unit of admission. The random sequence was generated by an independent statistician. Allocation is performed by the in-house research assistant using REDCap database only accessible by investigators. This database is password protected and will not be shared with those outside of study [14].

## Concealment mechanism

Providers and patients are not to be blinded to treatment allocation due to feasibility and cost. To address this issue, the opioid administration and pain score data (entered by nurses during routine clinical care) will be captured directly from the electronic medical record and the majority of outcomes will be obtained using our trauma registry (blinded outcome assessor).

## Analysis

The data analytic strategy will use Bayesian inference, applying generalized linear multilevel modeling with level-two random effects or fixed effects (depending on model convergence) to account for clustering of participants within department and, where applicable, observations within participants. Modeling will use R v. 3.4 and Stan v. 1.10 [15, 16]. For the purposes of evaluating the comparability of groups, a posterior probability that a difference/correlation exists of ≥ 95% will constitute evidence for statistically reliable differences. If potential confounders are identified, we will perform two sets of analyses: one in which the confounder(s) is included as a covariate and one in which it is not [17, 18]. This will permit determination of the degree to which any group differences might confound conclusions regarding treatment. All analyses will use intention-to-treat principles. Bayesian approaches will implement joint modeling of observed outcomes and the missing data which is robust to ignorable missingness (missing completely at random and missing at random) [19] Sensitivity analyses will evaluate robustness of analytic conclusions to missing data. Non-ignorable missing data patterns will be addressed through pattern-mixture modeling methods [20]. Convergence of Bayesian analyses on the posterior distributions via Monte-Carlo Markov chain (MCMC) will be assessed via graphical (Gelman-Rubin Plots) and quantitative (Gelman-Rubin Diagnostics and Effective Sample Size) evidence. Evaluation of posterior distributions will permit statements regarding the probability that effects of varying magnitudes exist, given the data. Diffuse, neutral priors will reflect the initial uncertainty regarding effect sizes. For all generalized linear multilevel models, priors for regression coefficients will be specified as ~Normal (*μ* = 0, *σ*^2^ = 1000) on the identity or log-scale depending upon the model, level one error variances will be specified as ~Half-T (df = 3, mean = 0, standard deviation = 100). Prior distribution for level two variances will use ~Half-T (df = 3, mean = 0, standard deviation = 1000). Priors for the comparison of proportions will be specified as ~Beta (*α* = 0.5, *β* = 0.5). For all subgroup analyses using multilevel models, the approach will follow that used in Tyson et al. [21].

## Data and safety monitoring board

As this is a comparative effectiveness trial utilizing ketamine at a lower dose than commonly used in the Emergency Department for moderate sedation during procedures (fracture reduction and splinting, tube thoracostomy, etc.), no data and safety monitoring board will be formed. Major adverse events, including death, will be reported to the Institutional Review Board (IRB).

## Data monitoring

Data is to be entered into RedCap for secure storage. REDCap is a mature, secure web application for building and managing databases. REDCap is compliant with standards such as HIPAA, 21 CFR Part 11, and FISMA (low, moderate, high). At UTHealth, REDCap is housed on two separate servers with both the database server and the web server located securely behind a firewall to improve confidentiality. Each item on the REDCap web forms will have validity checks performed to ensure that the data entered are accurate and that items are not skipped during entry by mistake. Depending on the question, any item found that does not meet the respective edit criteria will have an appropriate error message displayed when the user tries to save the data. Errors will be classified as either “hard” errors meaning that a valid response is required before the data can be saved or as “soft” errors in which the entry operator can either correct the errors or override them to indicate that the data are correct although it does not meet the edit criteria. Examples of hard errors would be items such as identifiers and event dates. An example of a soft error would be values that are outside a pre-defined range. Research personnel will have access to data for the purposes of data entry and data auditing. Investigators will have access to the data for purposes of data auditing and exporting. Once a record has been saved as complete, no one will be allowed to make changes to the records.

## Trial status

The IRB of McGovern Medical School approved the study protocol on September 30, 2019. Enrollment was delayed by the COVID-19 pandemic but began on July 9, 2020, and is scheduled to continue for 18 months. As of April 23, 2021, 193 patients have been enrolled.

## Discussion

Given that the current opioid epidemic was largely driven by legitimate opioid prescriptions after healthcare encounters, current acute pain management strategies must be thoughtful, responsible, and effective [4]. Emblematic of a learning trauma center, the KAPT trial builds on our work using a MMPR to provide adequate acute pain control in an opioid-minimizing manner with the ultimate goal of decreasing persistent opioid use.

The true rate of persistent opioid use following hospitalization for a traumatic injury is currently unknown, but trauma patients have often been considered at higher risk than general surgical patients. This higher risk has been attributed, in part, to opioid exposure during acute hospitalization and a larger proportion of patients who are considered “high risk” for persistent opioid use by the Opioid Risk Tool [22]. In elective surgery settings, strategies to combat the risk for persistent abuse have focused on enhanced recovery after surgery protocols, minimally invasive surgical advances, and evidence-based prescribing of opioids at the time of discharge [23]. The unplanned nature of injury largely precludes many of these interventions that have been so successful in elective surgery.

Our work in trauma patients has shown that reducing in-hospital opioid exposure leads to reduced prescriptions at discharge. In 2013, the median MME/day utilized at our institution prior to transition to MMPR was 65 (IQR 38, 90) and 81% of patients were discharged with an opioid prescription. In 2019, after universal implementation of generic MMPR, the median MME/day was 34 (IQR 15, 61), and 62% of patients were discharged with an opioid.

The KAPT trial is innovative in two main ways. First, it focuses on the first 72 h after admission, which we have shown is the time of highest opioid exposure after injury [5]. While a previous trial found no benefit to the addition of ketamine in patients with rib fractures, the authors did find a reduction of MME in the most severely injured subset [24]. The KAPT trial, in contrast, uses ketamine in a pragmatic manner in a heterogeneous patient population. The trial should answer the question: Does the routine addition of a sub-dissociative ketamine infusion decrease opioid exposure with acceptable acute pain control?

Second, the KAPT trial will be contacting patients after discharge to obtain 6-month follow-up information. Data regarding long-term pain and opioid use after injury is limited. We plan to determine the rate of persistent opioid use and persistent pain at 6 months following discharge.

## Limitations

One limitation of this study is that prehospital ketamine administration is not recorded. Since we are striving for adequate randomization, prehospital use should be equal among both groups and thus the impact of this should be minimal. An additional limitation is lack of blinding to treatment groups but this was necessary due to logistical restrictions.

In summary, the KAPT trial utilizes knowledge obtained from our previous work with opioid-minimizing regimens for traumatic pain and attempts advance our ability to decrease in-hospital opioid exposure and reduce opioid prescribing practices at discharge. In addition, this trial will enhance our understanding of long-term persistent opioid use, persistence of acute pain, and quality of life after injury. We hope to illustrate the safety of this drug and enhance our own learning healthcare system by allowing utilization of this medication across all levels of patient care and incorporating ketamine into our standard MMR.

## Data Availability

All data generated or analyzed during this study are included in this published article and its supplementary information files.
